# Seasonal Exposure to Drought and Air Warming Affects Soil Collembola and Mites

**DOI:** 10.1371/journal.pone.0043102

**Published:** 2012-08-15

**Authors:** Guo-Liang Xu, Thomas M. Kuster, Madeleine S. Günthardt-Goerg, Matthias Dobbertin, Mai-He Li

**Affiliations:** 1 Key Laboratory of Vegetation Restoration and Management of Degraded Ecosystems, South China Botanical Garden, Chinese Academy of Sciences, Guangzhou, China; 2 Forest Dynamics, Swiss Federal Research Institute WSL, Birmensdorf, Switzerland; 3 Long-term Forest Ecosystem Research, Swiss Federal Research Institute WSL, Birmensdorf, Switzerland; 4 Tree Physioecology, Swiss Federal Research Institute WSL, Birmensdorf, Switzerland; University of Otago, New Zealand

## Abstract

Global environmental changes affect not only the aboveground but also the belowground components of ecosystems. The effects of seasonal drought and air warming on the genus level richness of Collembola, and on the abundance and biomass of the community of Collembola and mites were studied in an acidic and a calcareous forest soil in a model oak-ecosystem experiment (the Querco experiment) at the Swiss Federal Research Institute WSL in Birmensdorf. The experiment included four climate treatments: control, drought with a 60% reduction in rainfall, air warming with a seasonal temperature increase of 1.4°C, and air warming + drought. Soil water content was greatly reduced by drought. Soil surface temperature was slightly increased by both the air warming and the drought treatment. Soil mesofauna samples were taken at the end of the first experimental year. Drought was found to increase the abundance of the microarthropod fauna, but reduce the biomass of the community. The percentage of small mites (body length 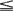 0.20 mm) increased, but the percentage of large mites (body length >0.40 mm) decreased under drought. Air warming had only minor effects on the fauna. All climate treatments significantly reduced the richness of Collembola and the biomass of Collembola and mites in acidic soil, but not in calcareous soil. Drought appeared to have a negative impact on soil microarthropod fauna, but the effects of climate change on soil fauna may vary with the soil type.

## Introduction

Soil mesofauna exert strong regulatory control over the soil food web and have substantial effects on important soil characteristics, including the distribution of soil particles, the soil’s water-holding capacity and water infiltration rate, the lability of organic compounds and mineralization, immobilization, the availability of N and other nutrients, the transport of compounds, and the composition, abundance, dispersal, and activity of bacteria and fungi [1]–[4]. Soil mesofauna are also particularly sensitive to environmental changes, and therefore thought to be an excellent bioindicator [5]–[7]. However, few studies on soil mesofauna have been made in the context of environmental change [8], in spite of its important ecosystem functions. This contrasts with the way above-ground effects [9], responses of soil microbial communities [10], litter decomposition [11], and nutrient cycling [12] have been extensively investigated. Environmental changes, such as changes in precipitation and air temperature, are likely to lead to changes in the mesofauna and could alter belowground biological processes, with potential consequences for ecosystem functions.

Warming and changes in precipitation amounts can strongly influence microarthropod reproduction and development rates by altering the soil temperature and moisture [8]. Soil moisture has often been reported to be the most important environmental variable affecting both the structure and function of the soil fauna community [13]–[z16]. Generally, it seems that the abundance of all faunal groups decreases with drought. For example, soil mites, one of the most abundant group of mesofauna has been found to be positively related to soil moisture across many ecosystems [17]–[20]. Drought seems to be a limiting factor for Collembola. For example, drought periods were associated with a reduction (in absolute numbers and diversity) in Collembola species that dwell in the forest litter and the moss layer [6], [14], [21]. Moisture-induced shifts in the community composition of soil mesofauna may influence community structure, which in turn may affect body size distribution, although to our knowledge, this has not yet been explicitly explored. These changes may have important impacts on ecosystem functions, including changes in decomposition rate under future climatic changes [8].

Warming can alter the soil fauna community by leading to changes in the abundance and composition of soil bacteria and fungi, and influencing plant physiology and community structure. Changes in community structure are directly related to the resource availability and microhabitat conditions of the ecosystem [9], [22]. According to several studies, the responses of soil fauna to warming vary with climate [8], [23]–[27]. Wolters [28] found that, for ten consecutive years, the annual mean temperature was significantly correlated with alterations in collembolan density in a beech forest on limestone (Göttingen, Germany). Oribatida and predatory mites (Mesostigmata and Prostigmata), in particular the Oribatida species *Diapterobates notatus*, also tended to increase in number with warming [25]. However, the abundance of most Collembola, including *Hypogastrura tullbergi*, *Lepidocyrtus lignorum* and *Isotoma anglicana*, tended to reduce with warming [25]. Only minor changes in the soil fauna occurred at higher temperatures, even after 6 years of elevated temperature treatment [29]. These contradictory results may be due to the significant side-effects of drought, which have often been found to accompany experimental increases in temperature. Harte et al. [1] found that warming increased microarthropod abundance and biomass only under wet conditions, but not under dry conditions. In fact, increases in temperature are often associated with decreases in moisture, and the indirect effects of changes in soil moisture may be more important for the survival and reproductive ability of soil fauna than the direct effects of warming [30]. Thus targeted experiments and monitoring studies are needed to distinguish between the effects of warming and drought.

The current study was performed as part of the “Querco” experiment [31], in which combined air warming and/or drought treatments were studied in two types of forest soils. The overall goal of the Querco experiment is to understand the effects of climate change on near-natural oak model ecosystems at the Swiss Federal Research Institute WSL [32]. We hypothesized that the abundance and biomass of soil Collembola and mites, and the taxon richness of Collembola, will be decreased by drought but increased by warming without interaction.

## Methods and Materials

### Design of the “Querco” Experiment

The current study was conducted in 2007 at the Swiss Federal Research Institute WSL in Birmensdorf, Switzerland, as part of the Querco experiment [31]. The experimental set-up included 16 hexagonal model ecosystem chambers (6 m^2^ in area, 3 m in height, and 1.5 m in soil depth), with roofs that close automatically during rainfall. The chambers were arranged in a Latin square with four treatments and four replications each. The treatments were control, air-temperature warming, drought, and air warming + drought. In the control treatment, opened chamber walls provided the same air temperature as at ambient conditions. In the air warming treatment, the opening of the chamber walls was reduced, which increased the air temperature in summer 2007 (June, July and August) passively by 1.4°C, in line with the IPCC A2 scenario for Switzerland [33]–[34]. Subsequently, soil temperature (−10 cm) was also increased, e.g. by 0.7°C in August 2007.

**Figure 1:**
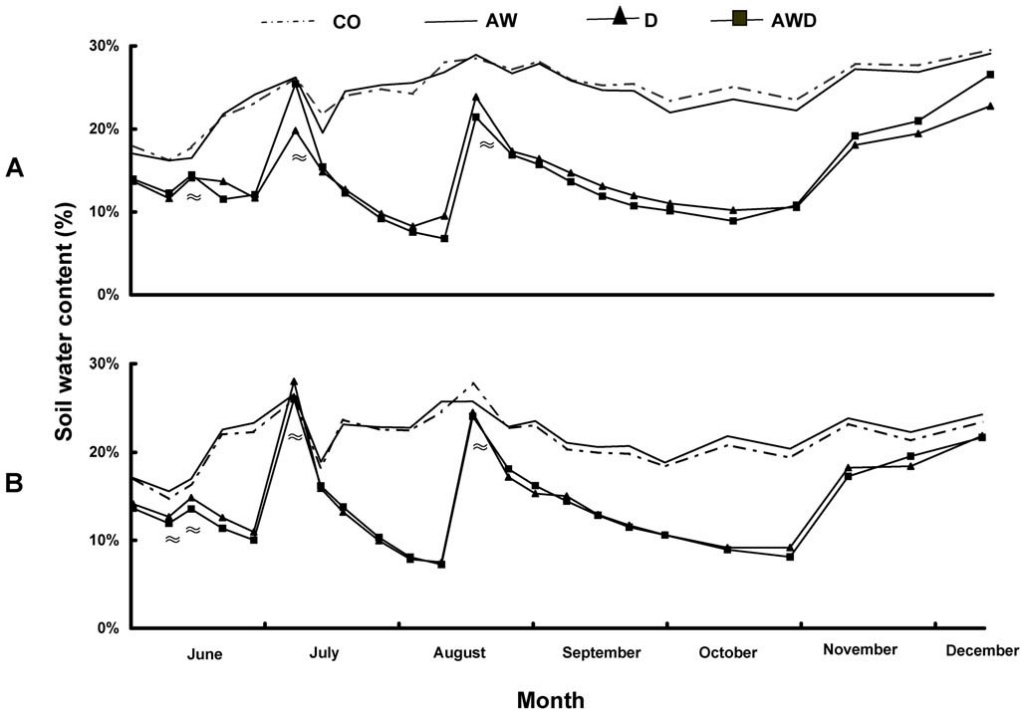
Effects of climate treatments and soil types on soil water content. Soil water content (SWC) at a depth of 0–25 cm in acid (A) and calcareous (B) soils as affected by the treatments: CO = control, AW = air warming, D = drought, and AWD = air warming + drought. Values are means of four plots in 2007. ≈ indicates no significant drought effects between (CO and AW) and (D and AWD) (n = 4).

Irrigation was applied with the same ion composition as the 30-year precipitation mean of the site [31]. The irrigation regime was the same in the air-warming treatment as in the control. Sufficient water (according to the monitoring by TDR and tensiometers) was supplied by means of sprinklers at intervals of 2 to 3 days from May to October. In the drought and air-warming + drought treatment, the irrigation was interrupted several times (Fig. 1). Therefore, in these treatments, the amount of irrigation from April to October was 60% lower than the long-term mean at the site, as it used as a reference to the severe variation in the IPCC A2 scenario. Each chamber was divided into two soil-lysimeter compartments containing one of two forest soils with similar soil texture, namely, acidic loamy sand (haplic alisol, pH 4.1) or calcareous sandy loam (calcaric fluvisol, pH 7.3). The sandy forest soils (for details, see Kuster et al. [31]) were taken in autumn 2005 from sites stocked with adult oaks, and then passively homogenized during transport and filling in the lysimeter compartments. In spring 2006, 24 2-year-old oak seedlings were planted in each soil compartment. Three common European oak species were selected, each with four provenances [32]. The oak seedlings were randomly distributed in each compartment. Treatments started in spring 2007 with the mesofauna samples taken at the end of the season.

### Determination of Soil Water Content, Soil Temperature, Foliage and Root Biomass, and Soil Respiration

Volumetric soil water contents were measured using time domain reflectometry (TDR 100, Campbell Scientific Inc., USA) in each soil compartment at 0–25 cm depth at 1-week intervals throughout the growing season from June to December 2007. Soil temperatures were measured hourly below the soil surface at a depth of 1 cm with iButton temperature loggers (Maxim Integrated Products Inc., USA). Foliage biomass was sampled at the end of the growing season in 2007. The root biomass in 2007 could not be measured as the experiment was running afterwards for another two years. We therefore estimated the root biomass in 2007 by using the allomectric relationship between foliage and root mass in 2009 and the foliage biomass sampled in 2007. The soil respiration rate was measured at permanent docking cylinders (diameter = 10 cm) in each soil subplot using a 6400-09 soil CO_2_ flux chamber connected to an LI-6400 infrared gas analyser (both LI-Cor Bioscience Inc, Lincoln, Nebraska, USA). Each measurement was conducted three times in a row to average out short-term variations.

### Sampling, Extraction and Identification of Soil Collembola and Mites

Soil cores (0–5 cm depth, 5 cm diameter) were collected from 22 October to 10 November 2007 with a steel cylinder at three locations in each soil compartment of three of the four replicate chambers. The three soil cores from each soil compartment in each chamber were combined to form a mixed sample. Immediately after collection, the soil samples were transported to the laboratory, and soil Collembola and mites were extracted using Tullgren dry funnels [35] for 48 hours. All specimens were sorted and counted with a dissecting microscope and examined with an Olympus BX41 research microscope. All Collembola were identified to genus level, mainly according to the keys in “Checklist of the collembolan of the world” [36], but also according to the keys in Potapov [37] and Bretfeld [38]. Abundance was expressed as ind. m^−2^. Soil mites were classified into four groups, namely Mesostigmata, Oribatida, Prostigmata and Astigmata [39].

### Biomass Calculations

Individual body length and width was measured at 10–80× magnification with a dissecting microscope equipped with an ocular micrometer with 0.01-mm precision. Dry biomass was calculated from regression equations estimating weights from linear dimensions:

Collembola dry mass: 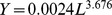 [40] (1)

where *Y* is the dry weight (mg) and *L* is the length (mm) of individuals.

The dry mass of the mites was calculated using the equations of Douce [41]:

Oribatida with *W_G_* × *W_B_ >*0.013: 
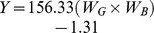
 (2)

Oribatida with *W_G_* × *W_B_ <*0.013: 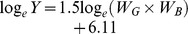 (3)

Mesostigmata: 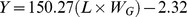 (4)

Prostigmata: 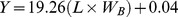
(5)

where *Y* is the dry weight (µg); *L* is the maximum body length (mm) excluding chelicera; *W_G_* is the maximum gnathosomal width (mm); and *W_B_* is the maximum body width (mm).

A few Astigmata were also found. Their body shape is similar to *Stigmaeus* (Prostigmata), and their biomass was estimated using equation (5).

### Statistical Analysis

Our experiment had a split-plot design with two subplots (soil types) within each plot (chamber with climate treatment). The main and interactive effects of the climate treatments and soil types were analyzed with an ANOVA accounting for split plots. The effect of the chambers was tested as a third factor, and climates were accordingly tested against its interaction with the chambers. The effect of the chambers was only kept in the ANOVA model if it was significant [42]. The differences among climate treatments in each soil type were evaluated with an LSD *post hoc* multiplied comparison. Pooling both soil types, the main and interactive effects between drought and warming were analyzed with a two-way ANOVA. The homogeneity of variances was confirmed by Levene’s test before analysis. All tests were considered to be significant at *P*<0.05 level. SPSS 13.0 was used for all analyses.

## Results

### Soil Water Content and Temperature

**Figure 2:**
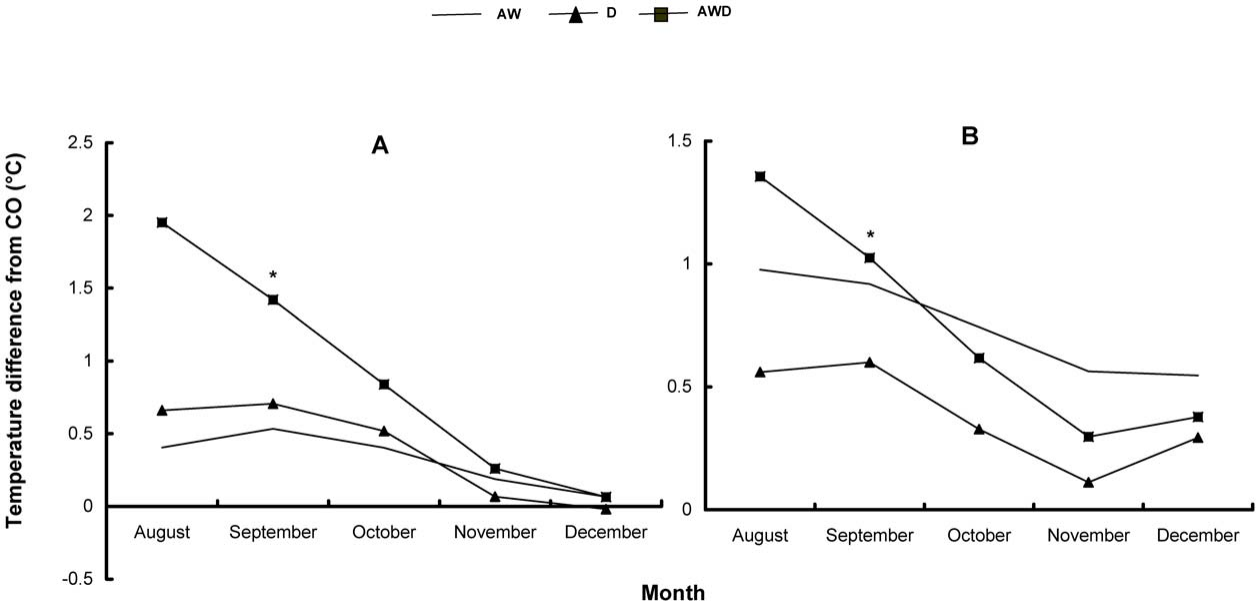
Effects of climate treatments and soil types on soil surface temperature. Mean surface temperature relative to controls (CO) in acid (A) and calcareous (B) soils as affected by the treatments: AW = air warming, D = drought, and AWD = air warming + drought in 2007, where * indicates a significant difference from CO (*P*<0.05, n = 4).

During the experiment, soil water content (SWC) did not differ between the air warming treatment and the control, and also not between drought and air warming + drought. This confirms that the temperature treatment was not confounded by drought effects (Fig. 1). SWC was substantially lower in soils, both acidic and calcareous, with a drought treatment (drought and air warming + drought) compared to in well-watered soils (control and air warming). After the plots were re-watered in July and August, the differences between the treatments disappeared accordingly (Fig. 1). There was a significant soil type effect on the SWC from mid August until the end of 2007, which indicates that the values in the acidic soil type were higher than in the calcareous soil type, especially in the control and air warming treatments. There was a positive air warming effect on the soil temperature in August, September and October, whereas the drought treatment raised the temperature below the soil surface only in August and September. However, when Tukey HSD pair-wise comparisons were performed for each soil type separately, the only significant difference was between control and air warming + drought in September (Fig. 2). There was no soil type effect on the monthly soil temperature.

### Plant Foliage, Root Biomass and Soil Respiration

The foliage biomass at the end of the 2007 growing season was significantly reduced by the drought treatment in the acidic soil type, but not in the calcareous soil type. A significant soil type effect indicated a higher foliage biomass and coarse root biomass in the calcareous than in the acidic soil type. Drought significantly reduced the coarse root biomass more in the acidic soil than in the calcareous soil (Table 1). As an indicator for soil respiration in 2007, the soil respiration at the end of a drought period in 2009 was significantly reduced by the drought treatment.

**Table 1:** Effects of climate treatments and soil types on soil respiration, foliage biomass and coarse root biomass.

		DW Foliage 07	DW Root 07	Soil Respiration
A	Control	0.156^a^ (0.013)	0.814^a^ (0.111)	6.9^a^ (0.6)
	Air-warming	0.121^ab^ (0.009)	0.531^a^ (0.058)	7.8^a^ (1.1)
	Drought	0.110^b^ (0.005)	* 0.526^a^ (0.037)	2.7^b^ (0.1)
	AW & D	0.119^ab^ (0.006)	0.555^a^ (0.036)	2.0^b^ (0.1)
B	Control	0.162^a^ (0.011)	0.835^a^ (0.058)	9.5^a^ (0.7)
	Air-warming	0.140^a^ (0.004)	0.697^a^ (0.021)	7.2^a^ (0.7)
	Drought	0.141^a^ (0.010)	* 0.822^a^ (0.051)	3.0^b^ (0.9)
	AW & D	0.126^a^ (0.005)	0.600^a^ (0.038)	2.8^b^ (0.5)

Air-warming (AW) and drought (D) treatment effects on soil respiration on 20.8.09 at the maximum of a drought period (µmol CO_2_ m^−2^ s^−1^), with the foliage biomass and coarse root biomass in 2007 (kg m^−2^, means ± SE) in the two soils (“A” acidic, “B” calcareous). Different letters indicate significant differences between the respective treatments in the same soil. DW indicates dry weight. An asterisk (*) indicates a significant difference between acidic and calcareous soil for the respective treatment.

### Abundance, Group Richness, and Biomass of Soil Collembola and Mites

**Figure 3:**
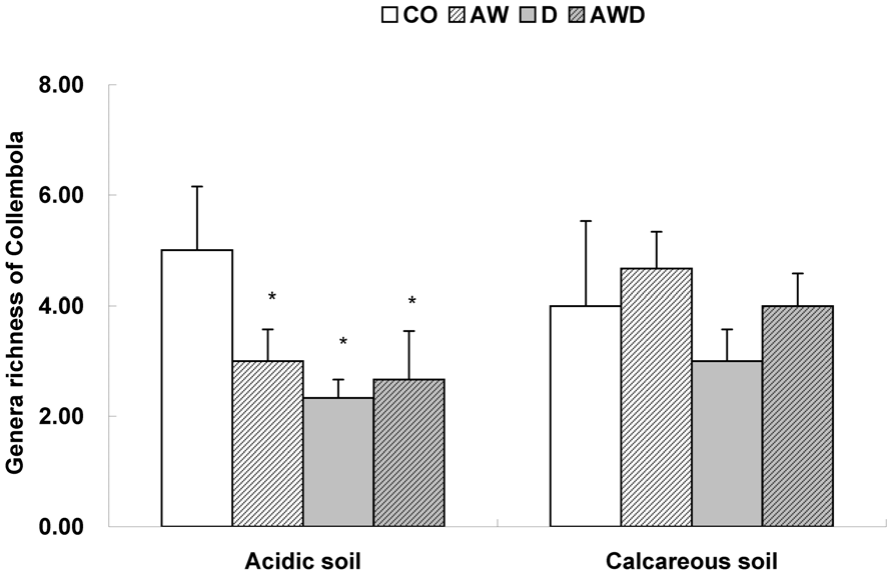
Effects of climate treatments and soil types on Collembola richness. Effects of the treatments (CO = control, AW = air warming, D = drought, and AWD = air warming + drought) on the genus level richness (number of genera) of Collembola in two forest soils (acidic and calcareous) in the Querco experiment at WSL in 2007, with mean values + SE, where * indicates significant differences from CO (*P*<0.05, n = 3).

**Figure 4:**
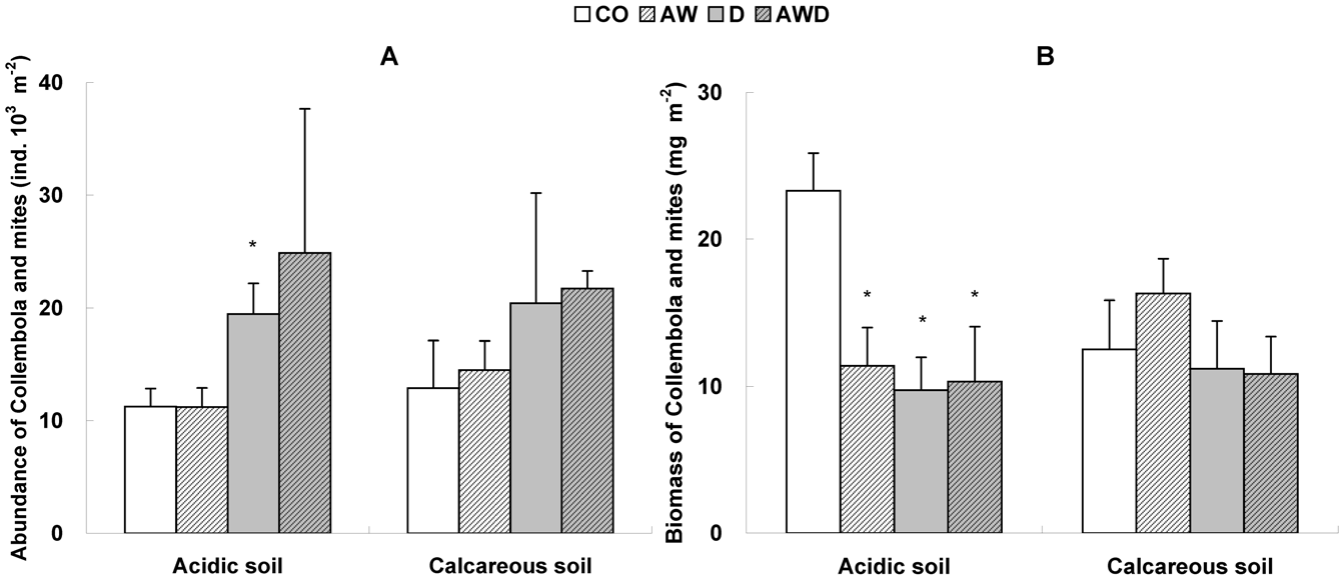
Effects of climate treatments and soil types on the community of Collembola and mites. Effects of the treatments (CO = control, AW = air warming, D = drought, AWD = drought + air warming) on (A) the abundance of Collembola and mites (ind. 10^3^ m^−2^) and (B) the biomass of Collembola and mites (mg m^−2^) in two forest soils (acidic and calcareous), with mean values + SE, where * indicates significant differences from CO (*P*<0.05, n = 3).

Fourteen genera of Collembola were identified, and the abundance of each Collembola genus and mite group in the four treatments and two soils were determined (Table 2). Only in the acidic soil, Collembola genus level richness was significantly reduced by all climate treatments (Fig. 3). There was a slight trend for Collembola to be richer in the calcareous than in the acidic soil type, with a weak climate-soil interaction (Table 3). Similarly, climate treatments reduced the biomass of the microarthropod fauna only in the acidic soil (Fig. 4B), and also with a weak climate-soil interaction (Table 3). If both soil types were combined, drought appeared to increase the abundance of microarthropod fauna (Table 4, Fig. 4A), but significantly to decrease their biomass (Table 4). This however, is probably mainly due to the effect of drought observed in the acidic soil (Fig. 4B).

**Table 2:** Abundance of soil Collembola and mites sampled in the Querco experiment.

	CO		AW		D		AWD	
	A	B	A	B	A	B	A	B
**Collembola**								
*Entomobrya*	57 (57)	–	–	–	–	–	–	–
*Folsomia*	57 (57)	–	57 (57)	–	–	57 (57)	–	–
*Isotomiella*	226 (150)	226 (226)	113 (113)	–	–	–	57 (57)	–
*Isotomodes*	–	–	–	57 (57)	113 (113)	113 (57)	–	340 (98)
*Lepidocyrtus*	–	–	–	–	–	–	113 (113)	–
*Marcuzziella*	57 (57)	–	–	–	–	–	–	–
*Neelides*	170 (98)	57 (57)	–	340 (170)	–	–	–	–
*Oligaphorura*	57 (57)	–	–	113 (113)	–	–	–	113 (57)
*Pachyotoma*	57 (57)	–	–	–	–	–	–	–
*Parisotoma*	170 (98)	226 (57)	679 (259)	623 (463)	962 (591)	226 (226)	226 (150)	170 (98)
*Proisotoma*	–	57 (57)	–	113 (113)	113 (113)	–	57 (57)	–
*Protaphorura*	–	57 (57)	–	–	–	–	–	–
*Thalassaphorura*	113	396	226	1189	–	510	–	623
	(113)	(247)	(226)	(490)	–	(259)	–	(396)
*Tullbergia*	4529	2378	4360	4926	7757	2944	5492	3680
	(1076)	(196)	(1306)	(2247)	(2072)	(1429)	(884)	(932)
**Acari**								
Astigmata	–	–	–	–	57 (57)	–	396 (204)	–
Mesostigmata	3227	2944	1755	3001	2038	623	2491	1019
	(354)	(1630)	(575)	(1331)	(260)	(150)	(1501)	(98)
Oribatida	1982	5718	3284	2831	2887	4360	5435	8096
	(653)	(2237)	(2362)	(982)	(1348)	(3041)	(3229)	(3906)
Prostigmata	566	849	736	1302	5548	11607	9229	7643
	(113)	(392)	(204)	(558)	(2774)	(8319)	(7967)	(2666)

Mean (SE) abundance of Collembola and mites (ind. m^−2^) as affected by the treatments (CO = control,

AW = air warming, D = drought, and AWD = air warming + drought) in two forest soil types (“A” acidic, “B” calcareous) in the Querco experiment at WSL.

**Table 3:** Effects of climate treatments and soil types on Collembola and mites.

	Collembola richness		Community abundance		Community biomass	
	*F*	*P*	*F*	*P*	*F*	*P*
Climates (*df* = 3)	1.00	0.44	1.45	0.30	2.66	0.12
Soils (*df* = 1)	3.05	0.12	0.03	0.87	0.26	0.62
Climates * Soils (*df* = 3)	2.41	0.14	0.11	0.95	3.18	0.09

*F*- and *P*- values of the main and interactive effects of treatments (climates) and soils (ANOVA, split-plot design in the Querco experiment at WSL) on Collembola genus level richness (number of genera), and the community (Collembola and mite) abundance and biomass.

**Table 4:** Effects of drought and air warming on Collembola and mites.

	Community		Community		Collembola of			Collembola of			Mite of		Mite of	
	abundance		biomass		small size			large size			small size		large size	
					(≤0.30 mm)			(>0.60 mm)			(≤0.20 mm)		(>0.40 mm)	
	*F*	*P*	*F*	*P*	*F*	*P*		*F*	*P*		*F*	*P*	*F*	*P*
Drought (*df* = 1)	5.62	0.05	6.18	0.04	0.03		0.86	3.76		0.09	20.51	<0.01	17.85	<0.01
Warming (*df* = 1)	0.26	0.63	0.84	0.39	0.23		0.64	0.12		0.74	1.52	0.25	0.34	0.57
Drought * Warming (*df* = 1)	0.04	0.85	0.94	0.36	<0.01		0.99	6.92		0.03	1.06	0.33	0.41	0.54

*F*- and *P*- values of the main and interactive effects of treatments (drought and warming) on the community (Collembola and mite) abundance and biomass, and on the number of Collembola of small size (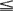 0.30 mm), Collembola of large size (>0.60 mm), mite of small size(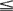
0.20 mm), and mite of large size(>0.40 mm) by two-way ANOVA in the Querco experiment at WSL.

### Body Size of Mites and Collembola as Affected by the Treatments

The body size distribution of soil mites was altered by drought (Table 4). Drought increased the percentage of mites with small body size (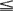 0.20 mm), but reduced the percentage of mites with large body size (>0.40 mm). The smaller body size was mainly to do with the effect of drought observed in the acidic soil (Table 5). Furthermore, the small and large mites responded differently to air warming. Smaller mites were generally negatively associated with air warming in both soil types, while larger mites were positively associated with air warming in calcareous soil (Table 5). The percentage of large Collembola (>0.60 mm) was reduced by drought in combination with warming (Table 4), whereas the soil type showed no distinguishable pattern (data not shown).

**Table 5:** Effects of climate treatments and soil types on body length categories of mites.

Length(mm)	Soil	Percentage of soil mites in each length category (%)			
		CO	AW	D	AWD
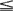 0.20	Calcareous	50 (18 ) ab	31 (14) b	84 (11) a	90 (5) a
	Acidic	40 (13) bc	33 (3 ) c	73 (2 ) a	67 (16 ) ab
0.21–0.40	Calcareous	39 (15) a	41 (23) a	8 (5) a	9 (5 ) a
	Acidic	8 (8 ) a	33 (14 ) a	14 (3 ) a	23 (12) a
>0.40	Calcareous	10 (3) b	28 (9 ) a	8 (6) b	1 (0) b
	Acidic	53 (6) a	34 (17) ab	12 (5 ) b	10 (6 ) b

The percentages of different body length of mites in each treatment and soil type in the Querco experiment at WSL. Values are means (SE). Values in a row followed by different letters are significantly different (*P*<0.05).

## Discussion

### Effects of Drought and Warming on Soil Collembola and Mites

Previous reports concerning the effect of warming on soil collembolan abundance have been inconsistent. This might be due to the inability of the previous experimental designs to distinguish the warming effects from the drought effects inadvertently caused by the warming treatment [6], [27]–[28]. In our study, the experimental design allowed us to separate the effects of warming and drought. The air warming treatment, which in the Querco experiment increased the air and the soil surface temperature slightly, only induced a negative response with respect to the richness of Collembola at the genus level and the biomass of Collembola + mites in the acidic soil (Fig. 3 and 4B). The effects on the soil water content of the two soils were similar (Fig. 1). Air warming not only had little effect on microarthropod fauna, apart from on their body size distribution, but it also had little effect on soil respiration, leaf biomass and coarse root biomass (Table 1).

In contrast, drought reduced the microarthropod biomass and Collembola richness, which is consistent with our hypothesis and with previous studies. Moisture conditions generally have a strong impact on Collembola behaviour and survival [13]. A field experiment in a spruce (*Picea abies*) monoculture forest near Giessen in Hesse, Germany, found that drought treatment drastically reduced the size of the Collembola communities in litterbags, and concluded that drought treatment stressed the Collembola, and reduced their abundance and species richness [14]. In a spruce forest in Sweden, euedaphic and hemiedaphic species of Collembola were distinguished and both were found to be negatively affected by experimental drought [21]. A long-term study (1992–2002) in Scots pine forests in the North Vidzeme Biosphere Reserve (northern Latvia) found that the abundance and diversity of Collembola species in the forest litter and the moss layer were reduced during drought periods [6].

How exactly drought affects Collembola is still not clear. Drought may, for example, directly influence the physiological reactions, resistance to dehydration, development responses, oviposition rate, or fecundity [21]. The indirect effects of drought may also play a role in determining microhabitat heterogeneity, fungi biomass and diversity. Soil bacteria and fungi are particularly sensitive components in soils, showing simultaneous responses to decreases in substrate humidity and quality [14]. The species structure of fungi is, for example, known to change with varying moisture levels [43]–[44].

Most Collembola and Oribatida are mycophagous and may selectively feed on different fungal species [5], [45]. For example, five needle-excavating oribatids species may need particular fungi to make oviposition possible [11]. A detailed analysis of the Collembola community structure showed that certain species are highly adapted to specific characteristics of the substrate and thus respond rapidly to changes in microhabitat conditions [14]. In the acidic soil in the Querco experiment, especially, such indirect effects have been partly confirmed by the way drought seems to reduce the fungal abundance (unpublished data) and soil respiration (Table 1). Changes in the soil fauna community also influence the functions of an ecosystem, e.g. decomposition [14] and nitrogen cycling [46].

In our study, we found that drought actually increased the abundance of the microarthropod fauna, which is inconsistent with some other reports [13], [20]–[21]. However, the contribution of mites to this result was difficult to estimate without specific taxonomic identification. For Collembola, we found that the significant reduction of richness caused by drought was mainly due to the absence of some rare genera, such as *Oligaphorura*, *Marcuzziella*, and *Pachyotoma*, and the increase in abundance under drought was dependent on the contribution of two genera, *Parisotoma* and *Tullbergia*, whose abundance increased by 200% and 55%, respectively, compared to that of the control. This might reflect the way soil fauna adopt different adaptation strategies to cope with low humidity, which may be morphological, physiological or reproductive adaptations [47].

Lindberg et al. [19] found some drought-tolerant Collembola species in the drought plots they studied using PCA analysis. A detailed analysis of the Collembola community structure indicated that certain species, such as *Willemia anophthalma* and *Mesaphorura tenuisensillata* seemed to be resistant to drought [14]. It could be that they reproduced through parthenogenesis, which had been shown to be a common strategy to increase the population among small euedaphic Collembola living deeper in the soil and among active colonizers, such as *Mesaphorura machrochaeta* (Familia: Tullbergiidae) and *Parisotoma notabilis* [48]. Our results indicated that the composition of Collembola species under drought tends to shift towards a dominance of drought-resistant species, while drought-sensitive species are at risk of disappearing.

### Effects of Drought on Body Size Distribution

Body mass is a fundamental organismal trait and is closely related to an organism’s physiology and ecology [49]. The body size distribution of soil mites was markedly changed by drought in this study, with an increase in small mites and a decrease in large mites. Whether this change in body size distribution resulted from a change in species composition or a change in size within species could not be determined. To our knowledge, few previous studies reported a change in the distribution of mesofauna body size in response to environmental changes. It seems, however, to be a general phenomenon in many animals, and reflects intraspecific change in some cases [50]–[52].

In Australia, light brown apple moths are reported to be smaller during warm, dry months than during cool, wet months [53]. Also in Australia, the growth and size of the wild brush-tailed phascogale is reduced during drought years [54]. Numerous authors have argued that such patterns of morphological variation are evidence of adaptation to environmental variables (referred to by Boyce [55]). Jones [52], for example, proposed that a small body size was advantageous during drought. However, a clear understanding of the adaptive significance of this variation is still missing. In a recent issue of the journal OIKOS, eight papers explored the influence of body size on many processes, ranging from individual biological rates to ecological networks [50]. Changes in body size reported here could imply changes in the soil food web, in the organism’s ecosystem functions, e.g. its metabolic or ingestion rate, and in its ecology. For example, its strength in interacting with other species, such as prey-handling ability and risk of being attacked by predators, may change, corresponding to changes in body size [49].

### Role of Soil Types

Our experiment showed that more changes as a result of the climate treatments occurred in the acidic than in the calcareous soil. The negative effects of drought on Collembola and mites in the acidic soil may be correlated with the presence of aluminum ions. Some soil fauna are reported to survive in stressed and polluted areas by using detoxification mechanisms, such as the activation of metal-binding proteins and the precipitation of metals as intracellular electron-dense granules [56]–[57]. However, the presence of aluminum ions is generally thought to be toxic. Aluminum ions have been found to be more mobilized in the acidic than in the calcareous soil (221 mg kg^-−1^ in the acidic vs. <2 mg kg^−1^ in the calcareous topsoil, Kuster, T.M., unpublished data) and can reach toxic levels for soil biota [12]. Another possible explanation for the strong effect of drought on mesofauna in the acidic than in the calcareous soil may relate to carbon, energy, and nutrient input for the food web. Drought significantly reduced the coarse root biomass in the acidic soil than in the calcareous soil (Table 1), and microbial biomass carbon and microbial biomass nitrogen were significantly lower in the acidic soil (Hu et al., personal communication). Roots, root exudates, decaying organic matter, and the associated microorganisms provide most of the carbon and energy that fuels the soil food web [58]–[59]. A reduction in these components may lead to a reduction in mesofauna richness.

The sensitivity of acidic soil to climate treatments, especially to drought, was also confirmed by the reduction in other components of the ecosystem, such as the coarse root and foliage biomass. For example, the foliage biomass was found to significantly decrease under drought in the acidic soil but not in the calcareous soil (Table 1). Over a three-year period the trees grew better on acidic than on calcareous soils, therefore consuming more water, these characteristics disappeared when drought was imposed, indicating stronger drought effects on trees grown in acidic than in alkaline soil [31]. Common beans are reported to grow longer roots and extract more soil moisture as an important mechanism to cope with soil water [60]. The roots of drought-tolerant bean species reached a soil depth of 1.3 m under drought stress at Palmira (soil pH 7.7), while the roots extended only to 0.7 m under acid soil conditions at Quilichao (soil pH 5.0) [60]. This suggests that the mechanism to cope with drought was inhibited in acidic soil. In conclusion, it seems that mesofauna are sensitive to air warming and drought, particularly in acidic soils.

## References

[1] Harte J, Rawa A, Price V (1996) Effects of manipulated soil microclimate on mesofaunal biomass and diversity. Soil Biology and Biochemistry 28: 313–322.

[2] Brussaard L (1997) Biodiversity and ecosystem functioning in soil. Ambio 26: 563–570. 10.1007/s13280-021-01507-z PMC8116420 33713290

[3] Freckman DW, Blackburn TH, Brussaard L, Hutchings P, Palmer MA, et al (1997) Linking biodiversity and ecosystem functioning of soils and sediments. Ambio 26: 556–562.

[4] Rusek J (1998) Biodiversity of Collembola and their functional role in the ecosystem. Biodiversity and Conservation 7: 1207–1219.

[5] Hopkin SP (1997) Biology of the Springtails (Insecta, Collembola). Oxford: Oxford University Press. 326p.

[6] Jucevica E, Melecis V (2006) Global warming affect Collembola community: A long-term study. Pedobiologia 50: 177–184.

[7] Xu GL, Schleppi P, Li MH, Fu SL (2009) Negative responses of Collembola in a forest soil (Alptal) under increased atmospheric N deposition. Environmental Pollution 157: 2030–2036. 19303182 10.1016/j.envpol.2009.02.026

[8] Kardol P, Reynolds WN, Norby RJ, Classen AT (2011) Climate change effects on soil microarthropod abundance and community structure. Applied Soil Ecology 47: 37–44.

[9] Kardol P, Cregger MA, Campany CE, Classen AT (2010) Soil ecosystem functioning under climate change: plant species and community effects. Ecology 91: 767–781. 20426335 10.1890/09-0135.1

[10] Castro HF, Classen AT, Austin EE, Norby RJ, Schadt CW (2010) Soil microbial community responses to multiple experimental climate change drivers. Applied Environmental Microbiology 76: 999–1007. 20023089 10.1128/AEM.02874-09 PMC2820983

[11] Hägvar S (1998) Mites (Acari) developing inside decomposing spruce needles: biology and effect on decomposition rate. Pedobiologia 42: 358–377.

[12] Matson P, Lohse KA, Hall SJ (2002) The globalization of nitrogen deposition: consequences for terrestrial ecosystems. Ambio 31: 113–119. 12077999 10.1579/0044-7447-31.2.113

[13] Verhoef HA, van Selm AJ (1983) Distribution and population dynamics of Collembola in relation to soil moisture. Holarctic Ecology 6: 387–394.

[14] Pflug A, Wolters V (2001) Influence of drought and litter age on Collembola communities. European Journal Soil Biology 37: 305–308.

[15] Tsiafouli MA, Kallimanis AS, Katana E, Stamou GP, Sgardelis SP (2005) Responses of soil microarthropods to experimental short-term manipulations of soil moisture. Applied Soil Ecology 29: 17–26.

[16] Moron-Rios A, Rodriguez MA, Perez-Camacho L, Rebollo S (2010) Effects of seasonal grazing and precipitation regime on the soil macroinvertebrates of a Mediterranean old-field. European Journal of Soil Biology 46: 91–96.

[17] Wallwork JA (1983) Oribatid in forest ecosystems. Annual Review of Entomology 28: 109–130.

[18] Badejo MA (1990) Seasonal abundance of soil mites (Acarina) in two contrasting environments. Biotropica 22(4): 382–390.

[19] Lindberg N, Engtsson JB, Persson T (2002) Effects of experimental irrigation and drought on the composition and diversity of soil fauna in a coniferous stand. Journal of Applied Ecology 39: 924–936.

[20] Badejo MA, Akinwole PO (2006) Microenvironmental preferences of oribatid mite species on the floor of a tropical rainforest. Experimental Applied Acarology 40: 145–156. 17103084 10.1007/s10493-006-9029-y

[21] Lindberg N (2003) Soil fauna and global change –Responses to experimental drought, irrigation, fertilisation and soil warming. Dissertation, Swedish University of Agricultural Sciences.

[22] Sjursen H, Michelsen A, Jonasson S (2005) Effects of long-term soil warming and fertilization on microarthropod abundances in three sub-arctic ecosystems. Applied Soil Ecology 30: 148–161.

[23] Coulson SJ, Hodkinson ID, Wooley C, Webb NR, Block W, et al (1996) Effects of experimental temperature elevation on high-arctic soil microarthropod populations. Polar Biology 16: 147–153.

[24] Huhta V, Hänninen SM (2001) Effects of temperature and moisture fluctuations on an experimental soil microarthropod community. Pedobiology 45: 279–286.

[25] Dollery R, Hodkinson ID, Jonsdottir IS (2006) Impact of warming and timing of snow melt on soil microarthropod assemblages associated with Dryas-dominated plant communities on Svalbard. Ecography 29: 111–119.

[26] McGeoch MA, Le Roux PC, Hugo EA, Chown S (2006) Species and community responses to short-term climate manipulation: Microarthropods in the sub-Antarctic. Austral Ecology 31: 719–731.

[27] Hägvar S, Klanderud K (2009) Effect of simulated environmental change on alpine soil arthropods. Global Change Biology 15: 2972–2980.

[28] Wolters V (1998) Long-term dynamics of a collembolan community. Applied Soil Ecology 9: 221–227.

[29] Haimi J, Laamanen J, Penttinen R, Räty M, Koponen S, et al (2005) Impacts of elevated CO_2_ and temperature on the soil fauna of boreal forests. Applied Soil Ecology 30: 104–112.

[30] Sinclair BJ, Stevens MI (2006) Terrestrial microarthropods of Victoria Land and Queen Maud Mountains, Antarctica: implications of climate change. Soil Biology and Biochemistry 38: 3158–3170.

[31] Kuster TM, Arend M, Bleuler P, Günthardt-Goerg MS, Schulin R (2012) Water regime and growth of young oak stands subjected to air warming and drought on two different forest soils in a model ecosystem experiment. Plant Biology 14: 1–10. 10.1111/j.1438-8677.2011.00552.x 22288508

[32] Arend M, Kuster T, Günthardt-Goerg MS, Dobbertin M (2011) Provenance-specific growth responses to drought and air warming in three European oak species (Quercusrobur, Q.petraea and Q.pubescens). Tree Physiology 31: 287–297. 21422189 10.1093/treephys/tpr004

[33] IPCC (2007) Climate Change 2007: Synthesis report. Cambridge University Press, Cambridge.

[34] CH2011 (2011) Swiss Climate Change Scenarios CH2011, C2SM, MeteoSwiss, ETH, NCCR Climate, and OcCC. CH2011, Zurich, Switzerland, 88.

[35] Brady J (1969) Some physical gradients set up in Tullgren funnels during the extraction of mites from poultry litter. Journal of Applied Ecology 6: 391–402.

[36] Janssens F (2007) Checklist of the Collembola of the world. http://www.collembola.org/.

[37] Potapov M (2001) Synopses on PalaearcticCollembola vol. 3. Isotomidae.Görlitz: State Saxonian Museum of Natural History. 603p.

[38] Bretfeld G (1999) Synopses on PalaearcticCollembola. vol. 2.Symphypleona.Görlitz: State Saxonian Museum of Natural History. 318p.

[39] Johnston DE (1982) Acari. In Synopsis and classification of living organisms, S.P. Parker (ed.), p. 111. McGraw-Hill, New York.

[40] Hόdar JA (1996) The use of regression equations for estimation of arthropod biomass in ecological studies. ActaŒcologica 17: 421–433.

[41] Douce GK (1976) Biomass of soil mites (Acari) in Arctic coastal tundra. Oikos 27: 324–330.

[42] Chen PY (2005) Statistics Software Tutorial of SPSS Application. People Medical Publishing House, Beijing.

[43] Bääth E, Söderström B (1982) Seasonal and spatial variation in fungal biomass in a forest soil. Soil Biology and Biochemistry 14: 353–358.

[44] Widden P (1986) Functional relationships between Quebec forest soil microfungi and their environment. Canadian Journal of Botany 64: 1424–1432.

[45] Maraun M, Migge S, Schaefer M, Scheu S (1998) Selection of microfungal food by six oribatid mite species (Oribatida, Acari) from two different beech forests. Pedobiologia 42: 232–240.

[46] Laakso J, Setälä H (1999) Sensitivity of primary production to changes in the architecture of belowground food webs. Oikos 87: 57–64.

[47] Alvarez T, Frampton GK, Goulson D (1999) The effects of drought upon epigeal Collembola from arable soils. Agricultural and Forest Entomology 1: 243–248.

[48] Chahartaghi M, Scheu S, Ruess L (2006) Sex ratio and mode of reproduction in Collembola of an oak-beech forest. Pedobiologia 50: 331–340.

[49] Digel C, Riede JO, Brose U (2011) Body sizes, cumulative and allometric degree distributions across natural food webs. Oikos 120: 503–509.

[50] Blanchard JL (2011) Body size and ecosystem dynamics: an introduction. Oikos 120: 481–482.

[51] Petchey OL, Belgrano A (2010) Body-size distributions and size-spectra: universal indicators of ecological status? Biology letters 6: 434–437. 20444761 10.1098/rsbl.2010.0240 PMC2936225

[52] Jones G (1987) Selection against large size in the Sand Martin Ripariariparia during a dramatic population crash. Ibis 129: 274–280.

[53] Danthanarayana W (1975) Factors determining variation in fecundity of the light brown apple moth, EpiphyasPostvittana (Walker) (Tortricidae). Australian Journal of Zoology 23: 439–451.

[54] Rhind SG, Bradley JS (2002) The effect of drought on body size, growth and abundance of wild brush-tailed phascogales (Phascogale tapoatafa) in south-western Australia. Wildlife Research 29: 235–245.

[55] Boyce MS (1978) Climatic variability and body size variation in the muskrats (Ondatrazibethicus) of North America. Oecologia 36: 1–19. 28309223 10.1007/BF00344567

[56] Dallinger R (1996) Metallothionein research in terrestrial invertebrates: synopsis and prospectives. Comp. Biochem. Physiol. 113 C: 125–133. 10.1016/0742-8413(95)02078-0 8646613

[57] Pigino G, Miglliorini M, Paccagnini E, Bernini F (2006) Localisation of heavy metals in the midgut epithelial cells of Xenillus tegeocranus (Hermann, 1804) (Acari: Oribatida). Ecotoxicology and Environmental Safety 64: 257–263. 16460803 10.1016/j.ecoenv.2005.12.012

[58] Norby RJ, Jackson RB (2000) Root dynamics and global change: Seeking an ecosystem perspective. New Phytologist 147: 3–12.

[59] Pollierer MM, Langel R, Körner C, Maraun M, Scheu S (2007) The underestimated importance of belowground carbon input for forest soil animal food webs. Ecology Letters 10: 729–736. 17594428 10.1111/j.1461-0248.2007.01064.x

[60] Sponchiado BN, White JW, Castillo JA, Jones PG (1989) Root growth of four common bean cultivars in relation to drought tolerance in environments with contrasting soil types. Experimental Agriculture 25: 249–257.

